# Environmental pollution’s toll on the heart: rethinking cardiovascular risk factors

**DOI:** 10.1093/ehjopen/oeae017

**Published:** 2024-03-02

**Authors:** Sameer Mehta, Yashendra Sethi

**Affiliations:** Department of Pollution Health, Lumen Foundation, 185 Shore Drive South, Miami, FL 33133​, USA; Department of Pollution Health, Lumen Foundation, 185 Shore Drive South, Miami, FL 33133​, USA

In the landscape of cardiovascular health, a paradigm shift is imperative as we move beyond traditional risk factors like smoking, hypertension, diabetes, and hyperlipidaemia (*Figure 1*). The contemporary nemesis emerges from the insidious clutches of environmental pollution, transcending borders and affecting populations on a global scale. Epidemiologists are increasingly focusing on non-traditional risk factors in built, natural, and social environments, significantly impacting disease burden and residual risk. These factors interact in complex, unpredictable ways, amplifying their effects. To address this, epidemiologists have introduced the concept of the ‘exposome’, encompassing the entirety of exposure to these novel risk factors.^1^ With this viewpoint, we underline the urgency for a recalibration of our understanding of cardiovascular risk factors, emphasizing pollution’s profound impact on cardiovascular, atherosclerotic, and cerebrovascular health.

**Figure 1:**
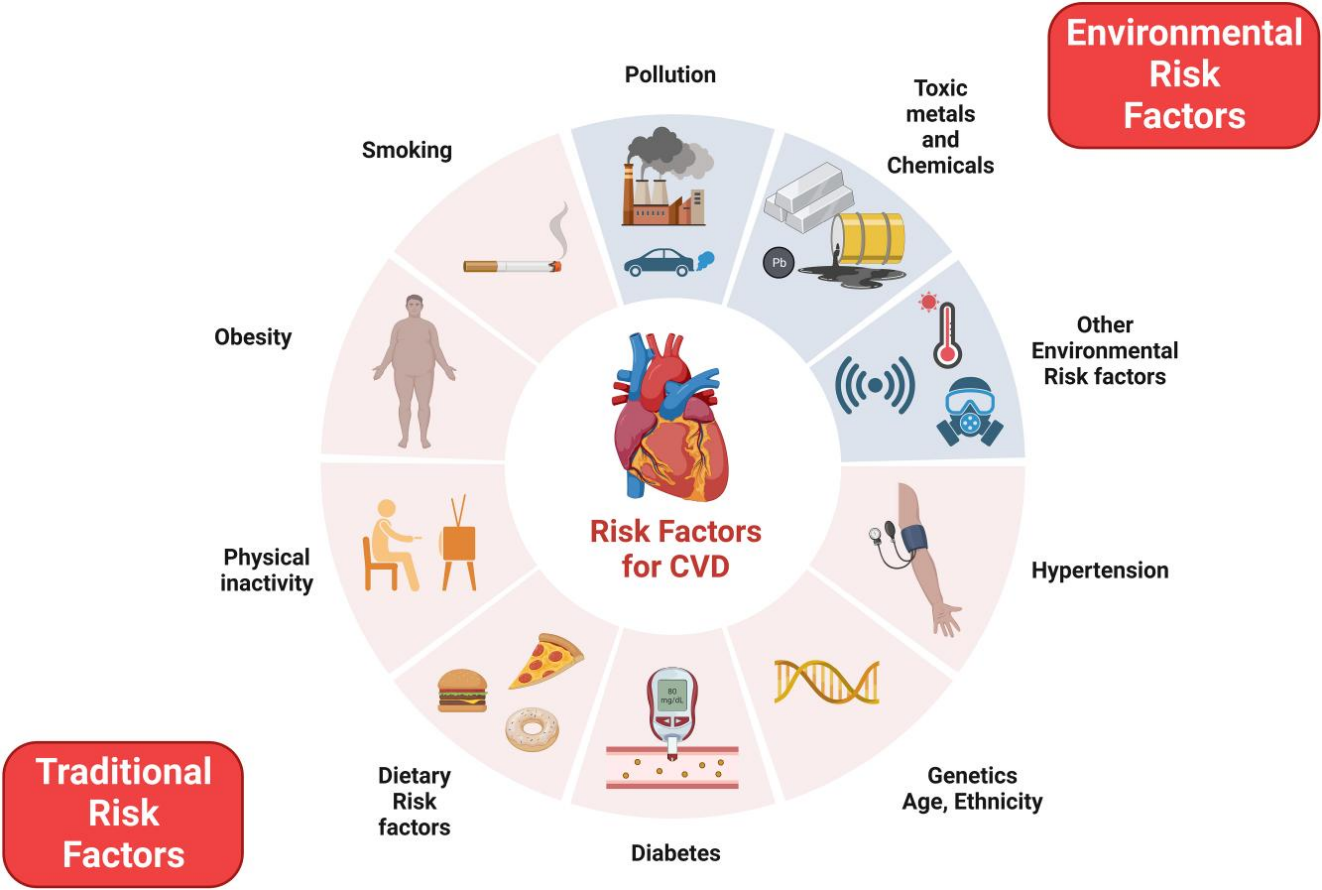
The traditional and evolving risk factors for cardiovascular diseases (CVD). Created with BioRender.com.

While the concept of ‘one health’ has gained traction for various infectious and lifestyle diseases, the spotlight on environmental factors for cardiovascular diseases (CVD) has regrettably missed the deserved attention. Cardiovascular disease prevalence nearly doubled from 271 million in 1990 to a staggering 523 million in 2019. Simultaneously, disability years surged from 17.7–34.4 million. It is high time we broaden our perspective to encompass the critical role of environmental pollution in the escalating prevalence and impact of CVD.^2^

The elucidation of CVD risk factors traces its roots to the seminal Framingham Heart Study, 1971. This pivotal research not only exposed lacunae in prevailing understanding of CVD but also identified salient risk factors, including hypertension, hypercholesterolaemia, smoking, obesity, diabetes, and sedentary behaviour. Furthermore, it furnished invaluable insights into the impact of ancillary determinants such as blood triglyceride and HDL cholesterol levels, age, gender, and psychosocial factors.^2^ Notwithstanding these advances, the intricate nature of CVD became apparent, with no solitary risk factor emerging as the exclusive aetiological agent; rather, a complex interplay of interconnected factors was implicated in its genesis. A noteworthy development in this trajectory was the belated recognition of environmental risk factors. In 2004, a pivotal AHA Scientific Statement disseminated by an expert consortium of 11 researchers and physicians underscored air pollutants as a ‘serious public health problem’ for CVD, marking a watershed moment for environmental cardiology. Less than two months subsequent to the AHA statement, the US Environmental Protection Agency accentuated the gravity of the matter by conferring its most substantial scientific research grant to date—amounting to $30 million—to investigate the interplay between air pollution and CVD. However, despite this acknowledgment, the exigency of addressing environmental risk factors has yet to receive commensurate scholarly attention. Regrettably, even after the passage of two additional decades, insufficient emphasis has been placed on elucidating and ameliorating the environmental determinants, while levels of pollution are continuously increasing.^1,3^

The overwhelming evidence suggests that pollution may now be a very significant ‘missed’ risk factor for cardiovascular health, contributing not only to reduced life expectancy but also to severe lung diseases and cancers. Low- and middle-income countries are anticipated to shoulder a disproportionate burden of CVD, primarily attributed to environmental factors. Among these factors, air pollution stands out, alongside exposure to various metals including arsenic, cadmium, lead, and other substances like per- and polyfluoroalkyl substances.^3^ The worsening air quality has seen a parallel increase in CVD even among population with no evident conventional risk factors—especially the young adults. Exposure to air pollution, particularly PM2.5, elevates the risk of myocardial infarction, stroke, heart failure, arrhythmia, and sudden death by 1–2% per 10 μg/m^3^ increase. Chronic exposure increases risk by 5–10%. Immediate oxidative stress and chronic inflammation, stemming from particle inhalation, affect cardiopulmonary and systemic vasculature, impacting all organ systems.^1–3^ In a major national capital like New Delhi, the pollution is projected to slash life expectancy by 10–12 years for every citizen, irrespective of traditional risk factors.

Pulmonologists and oncologists have traditionally prioritized environmental factors; however, it is pertinent to acknowledge the direct influence of pollution on CVD. Moreover, its capacity as an effect modifier for other risk factors necessitates scrutiny. Embracing a ‘one health’ paradigm in cardiology is imperative, as it allows for a holistic understanding of the intricate relationship. This awareness will help enhance our primary and secondary prevention strategies, leading to improved patient outcomes. It is incumbent upon researchers, academics, and policymakers to urgently reassess priorities. While we meticulously scrutinize traditional risk factors, the alarming truth remains: our entire planet is suffocating under the weight of pollution-induced health crises that indiscriminately affects everyone. Confronting the pollution should be healthcare priority #1, heralding a new era in safeguarding global cardiovascular health.

## Data Availability

No data were generated or analysed for or in support of this paper.
